# Delta rhythmicity is a reliable EEG biomarker in Angelman syndrome: a parallel mouse and human analysis

**DOI:** 10.1186/s11689-017-9195-8

**Published:** 2017-05-08

**Authors:** Michael S. Sidorov, Gina M. Deck, Marjan Dolatshahi, Ronald L. Thibert, Lynne M. Bird, Catherine J. Chu, Benjamin D. Philpot

**Affiliations:** 1 Department of Cell Biology and Physiology, University of North Carolina, Chapel Hill, NC 27599 USA; 2 Carolina Institute for Developmental Disabilities, University of North Carolina, Chapel Hill, NC 27599 USA; 3 Neuroscience Center, University of North Carolina, Chapel Hill, NC 27599 USA; 4 Department of Neurology, Massachusetts General Hospital, Boston, MA 02114 USA; 5 Harvard Medical School, Boston, MA 02215 USA; 6 Department of Pediatrics, University of California, San Diego, CA USA; 7 Division of Dysmorphology/Genetics, Rady Children’s Hospital, San Diego, CA USA; 8 Present Address: The Neurology Foundation, Rhode Island Hospital and Warren Alpert School of Medicine at Brown University, Providence, RI 02903 USA

**Keywords:** Angelman syndrome, Biomarker, Delta, EEG, Mouse model, Outcome measure, UBE3A

## Abstract

**Background:**

Clinicians have qualitatively described rhythmic delta activity as a prominent EEG abnormality in individuals with Angelman syndrome, but this phenotype has yet to be rigorously quantified in the clinical population or validated in a preclinical model. Here, we sought to quantitatively measure delta rhythmicity and evaluate its fidelity as a biomarker.

**Methods:**

We quantified delta oscillations in mouse and human using parallel spectral analysis methods and measured regional, state-specific, and developmental changes in delta rhythms in a patient population.

**Results:**

Delta power was broadly increased and more dynamic in both the Angelman syndrome mouse model, relative to wild-type littermates, and in children with Angelman syndrome, relative to age-matched neurotypical controls. Enhanced delta oscillations in children with Angelman syndrome were present during wakefulness and sleep, were generalized across the neocortex, and were more pronounced at earlier ages.

**Conclusions:**

Delta rhythmicity phenotypes can serve as reliable biomarkers for Angelman syndrome in both preclinical and clinical settings.

**Electronic supplementary material:**

The online version of this article (doi:10.1186/s11689-017-9195-8) contains supplementary material, which is available to authorized users.

## Background

Angelman syndrome (AS) is a neurodevelopmental disorder characterized by developmental delay, impaired speech and motor skills, and high comorbidity with epilepsy [1]. Loss-of-function mutations in the maternal copy of the imprinted *UBE3A* gene cause AS [2, 3], while maternal duplications in the same region (15q11-13) are linked to autism [4–6]. Recent work has identified multiple approaches with preclinical therapeutic potential for AS: antisense oligonucleotides and topoisomerase inhibitors have the potential to unsilence paternal *UBE3A* and re-express UBE3A protein; gene therapy provides a direct method of expressing *UBE3A*; mechanism-based approaches downstream of *UBE3A* include GABA_A_ agonists (THIP/gaboxadol) and modulation of αCaMKII; other approaches include altering diet [7–12]. Many of these approaches are in the pipeline for upcoming clinical trials. It is therefore critically important to develop biomarkers for AS that are clinically relevant, objectively quantifiable, highly penetrant, and have strong face validity between animal models and patient populations. Such biomarkers need not have predictive or diagnostic value, as AS diagnoses are confirmed genetically [13], but rather their value would lie primarily in their use as outcome measures.

Electroencephalography (EEG) has revealed consistent signatures of AS, which have been described by clinical reports and case studies spanning nearly 30 years [14–22]. EEG abnormalities in AS include rhythmic delta, rhythmic theta, and epileptiform spike-wave discharges. Increased delta rhythmicity is the most common EEG phenotype in AS (~84% of patients) [21], and of these phenotypes, it is the most specific for AS relative to other syndromes [20]. Multiple variants of delta activity have been described based on brain region and waveform characteristics [20], yet every variant of delta, by definition, has a common oscillation frequency of ~2–4 cycles per second. Clinical studies typically report delta abnormalities in a binary fashion, being present or absent, but in some cases have further subdivided delta abnormalities into being continuous or intermittent [17]. To date, no study has quantified delta rhythmicity in AS, quantitatively compared AS individuals to a neurotypical control group, or quantitatively tracked developmental and state-dependent (sleep/wake) changes in delta oscillations in AS. Principled characterization of these features, and validation in a mouse model, are critical for development of delta rhythms as a biomarker.

AS model mice (*Ube3a* ^*m−/p+*^) have genetic construct validity with the human condition and thus provide a powerful preclinical model. Silencing of the paternal *Ube3a* allele is conserved from humans to mice; thus *Ube3a* ^*m−/p+*^ mice, like individuals with AS, have minimal functional UBE3A protein [23]. Using parallel quantitative methods, we analyzed delta rhythmicity in AS model mice and human EEG data. We found that increased delta power provides a robust and reliable biomarker with strong face validity between the AS mouse model and a patient population, 4–11 years old. Additionally, quantitative methods allowed for a novel study of delta “dynamics,” a measure of how delta rhythms vary over time across a single recording session. Delta activity is more dynamic, both in AS mice and AS individuals. Children with AS exhibited enhanced delta activity across all EEG electrode placements. The enhanced delta power and dynamics were present during both wakefulness and sleep and were observed at all ages tested but most pronounced in younger children. Overall, this study corroborates qualitative clinical descriptions of delta oscillations in AS individuals [14–22], provides the first quantitative assessment of delta rhythmicity in AS individuals and comparison with a neurotypical reference group, and validates this biomarker in a mouse model. Delta rhythmicity thus has promise as a preclinical and clinical biomarker for AS and as an outcome measure for AS clinical trials.

## Methods

### Study design

**Fig. 1:**
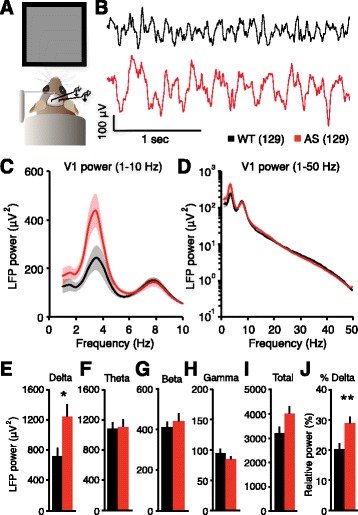
Delta power is increased in AS model mice. **a** LFP recording configuration from the primary visual cortex in awake mice. **b** Representative examples show increased delta rhythmicity in AS model mice. **c**, **d** Power spectra of group data (WT: *n* = 23, AS: *n* = 24; *shading* indicates ±sem; **d** is plotted on log scale). **e** Enhanced delta power (2–4 Hz) in AS mice (*p = 0.012, Student’s *t* test). **f** Theta (5–10 Hz), (**g**) beta (13–30 Hz), (**h**) gamma (30–50 Hz), and (**i**) total (1–50 Hz) power were not different between groups (theta: *p* = 0.858, beta: *p* = .509, gamma: *p* = 0.304, total: *p* = 0.075). **j** Enhanced relative delta power in AS mice (**p = 0.008)

**Fig. 2:**
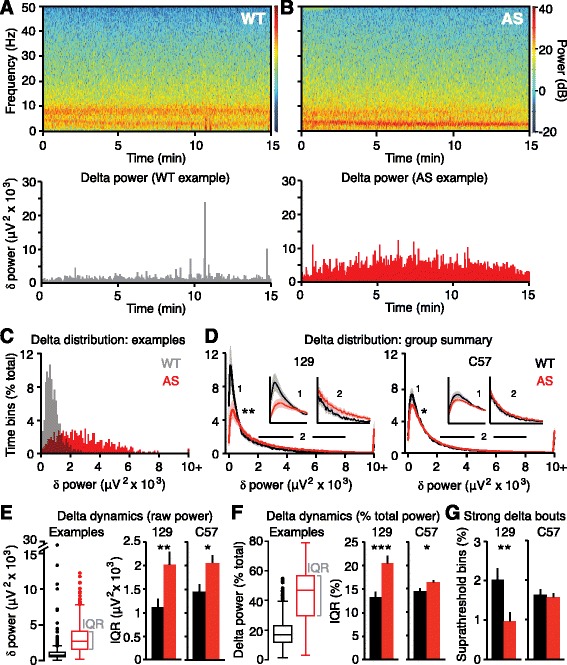
Delta rhythms are more dynamic in AS model mice. **a**, **b** *Top*: spectrograms show rhythmicity across time in single LFP sessions in representative WT and AS mice on a 129 background. *Bottom*: delta power is extracted from the spectrogram during each 2-s time bin for representative examples. **c** Distributions of delta power across a single session for representative examples used in **a** and **b. d** Group analyses reveal differences in delta distributions in both 129 and C57 strains (129: *p = 0.0005, C57: *p = 0.0198, K-S tests). *Inset 1* spans (in μV^2^ × 10^3^) 0–1 on the *x-axis*; *inset 2* spans 2–8. **e** Quantification of within-session delta dynamics. *Box plots* indicate representative examples used in **a** and **b**. Interquartile range (*IQR*) measures the spread of the middle 50% of delta measurements within a session. *Dots* represent suprathreshold bins where delta > Q3 + 1.5*IQR. Group analyses reveal increased IQR in AS model mice on both 129 and C57 backgrounds (129: **p = 0.0093, C57: *p = 0.010). **f** Calculating IQR based on relative delta power within each 2-s bin reveals AS model mice on both 129 and C57 backgrounds have increased within-session delta dynamics (129: ***p = 0.0008, C57: *p = 0.021). **g** There are fewer suprathreshold delta bouts in 129 AS model mice (**p = 0.007) but not C57 AS model mice (*p* = 0.74) compared to WT littermates

Our prespecified goal was to quantify delta power in AS: first in a mouse model, then in a patient population. We refined analysis methods during mouse studies and used these parameters for subsequent human EEG data analysis. We allowed for the possibility that mouse studies would shift our area of interest to other frequencies (e.g., theta) that have also been reported as abnormal in AS [14, 17, 20, 21, 24]; however, because mouse studies confirmed delta abnormalities with largely normal power in other frequency bands, we entered human studies with the original prespecified hypothesis that delta power is increased. We became interested in a secondary experimental question—the dynamics of delta abnormalities in AS—during mouse studies. Therefore, based on our mouse work (Figs. 1 and 2, Additional files 1: Figure S1 and 2: Figure S2) and clinical reports [14–22], we began human EEG studies with a clear hypothesis: delta power and dynamics are quantitatively increased in children with AS. We thus avoided problems with circularity and data selection that may arise when a study “fishes” for a phenotype with no predefined hypothesis [25].

**Fig. 3:**
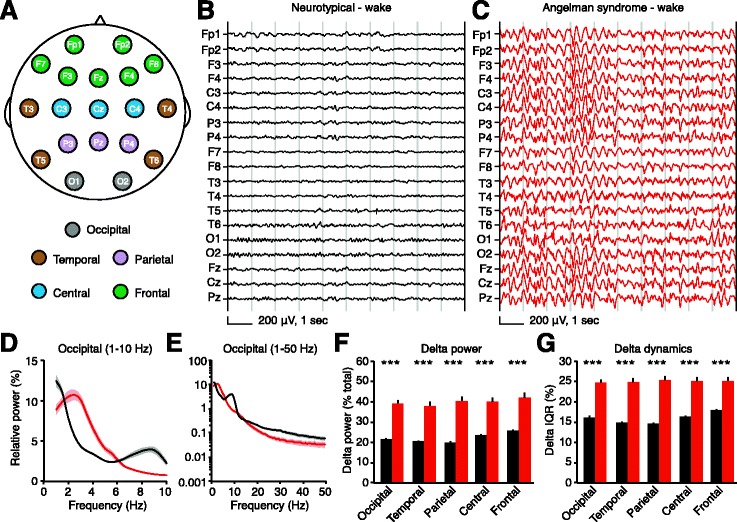
Delta rhythmicity is increased in children with Angelman syndrome relative to neurotypical controls during wakefulness. *Black*: neurotypical, *red*: AS. **a** Schematic showing EEG electrode placement according to the 10-20 recording system. Delta power and dynamics are calculated for each electrode and results averaged by region. Representative EEGs from **b** a neurotypical child and **c** a child with AS illustrate enhanced delta power, generalized across recording sites. **d**, **e** Power spectra of group data from occipital electrodes (NT: *n* = 54, AS: *n* = 26; *shading* indicates ±sem) illustrate an increase in delta power in AS; other regional spectra are shown in Additional file 3: Figure S3. **f** Group analyses reveal increased delta power generalizes across the neocortex (***p < 0.0001, Student’s *t* test). **g** Delta dynamics (IQR) are also increased in all regions (***p < 0.0001)

**Fig. 4:**
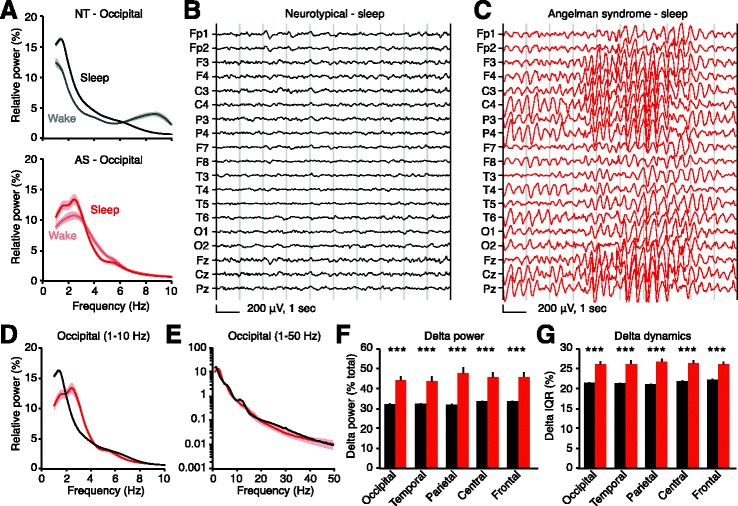
Delta rhythmicity is increased in children with Angelman syndrome relative to neurotypical controls during sleep. *Black*: neurotypical (NT), *red*: AS. **a** Occipital power spectra comparing wakefulness and sleep in neurotypical and AS children. Wake data are re-plotted from Fig. 3d; sleep data are re-plotted in **d**. Representative sleep EEGs from **b** a neurotypical child and **c** a child with Angelman syndrome illustrate delta oscillations in AS. **d**, **e** Occipital power spectra during sleep (NT: *n* = 54, AS: *n* = 13; *shading* indicates ±sem) show an increase in delta power in AS. **f** Group analyses reveal increased delta power generalizes across the neocortex (***p < 0.0001, Student’s *t* tests). **g** Delta dynamics (IQR) are also increased in all regions (***p < 0.0001)

**Fig. 5:**
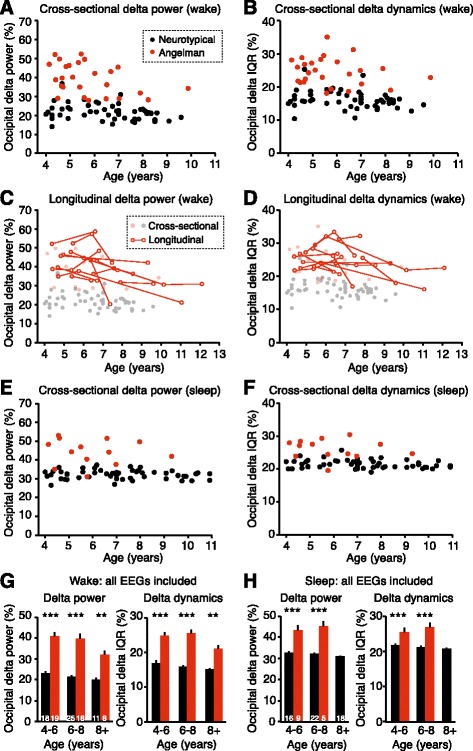
Delta phenotypes are stronger at earlier ages in children with Angelman syndrome. **a** Increased occipital delta power in children with AS is age-dependent during wakefulness (NT: *n* = 54, AS: *n* = 26). **b** Occipital delta dynamics as a function of age in neurotypical and AS children. Longitudinal studies in a subset of AS patients show that **c** delta power and **d** delta dynamics decrease as a function of age (*n* = 12 children, *n* = 31 sessions). **e** Delta power during sleep (NT: *n* = 54, AS: *n* = 13) and **f** delta dynamics during sleep do not show statistical age dependence. **g**, **h** Analysis of grouped cross-sectional and longitudinal occipital delta power and dynamics during wakefulness and sleep. **g** Delta power during wakefulness was increased in AS at ages 4–6, 6–8, and 8+ (two-way ANOVA and post hoc Bonferroni: ***p < 0.0001, **p = 0.0002). Delta dynamics (IQR) during wakefulness were increased in AS at ages 4–6, 6–8, and 8+ (***p < 0.0001, **p = 0.0007). Sample sizes are represented in *bars*. **h** Delta power and dynamics during sleep were increased in AS at ages 4–6 and 6–8 (***p < 0.0001)

Mouse studies were conducted on AS model mice (*Ube3a* ^*m−/p+*^) and wild-type littermate controls, with experimenters blind to genotype. Human studies were retrospective analyses of AS and neurotypical EEG data. Human subjects were children with a genetic diagnosis of AS who had EEGs between 2006 and 2014 at the San Diego site (Rady Children’s Hospital San Diego: RCHSD) of the AS Natural History Study (ClinicalTrials.gov identifier: NCT00296764), and an age- and sex-matched sample of neurotypical controls who had EEGs at Massachusetts General Hospital (MGH) between February 1, 2012, and May 1, 2012. For neurotypical EEGs, clinical chart review was performed and only those children with documented normal neurodevelopment and events leading to diagnostic EEG evaluation that were subsequently determined to be nonepileptic were included for analysis. We analyzed EEGs from children aged 4–11 (48–132 months), as this period is relatively stable compared to earlier ages [26] and is a likely age range for clinical trials. For cross-sectional studies (Figs. 3, 4, and 5a, b, e, f, Additional file 3: Figure S3), we analyzed one EEG session per subject. We followed up with longitudinal studies in a subset of children where multiple EEG sessions were available (Fig. 5c, d). In the longitudinal group, we analyzed two EEGs outside of our initial age parameters, both from children aged 11–12.

Because delta rhythms are a feature of slow-wave sleep and AS individuals have abnormal sleep patterns [1, 27], sleep during EEG sessions was a potential confounding variable. Therefore, an experienced clinical neurophysiologist manually categorized wake and non-REM sleep epochs using standard criteria [28]. We made AS versus neurotypical comparisons separately for periods of sleep and periods of wakefulness.

Our mouse sample size was determined a priori. Our retrospective human EEG sample sizes (wake and sleep) were determined by availability of de-identified data. All data exclusion criteria were defined prospectively for both mouse and human studies. For mouse studies, individual sessions were excluded (blind to genotype) only in rare cases where a headcap detached during a recording session or where movement artifacts were continuous and pervasive. Outliers in which total raw power (1–50 Hz) exceeded 2 SD from the group mean were excluded (number of outliers per group—129/WT, 2/25; 129/AS, 0/24; C57/WT, 1/31; C57/AS, 1/40). For human studies, individual electrodes were excluded in cases of excessive noise or poor connections. EEG recordings with less than 100 s of sleep or wake were excluded [29].

### Mouse LFP methods

#### Animals

All mouse protocols were approved by the Institutional Animal Care and Use Committee of the University of North Carolina at Chapel Hill. Mice were group-housed on a 12:12 light/dark cycle with ad libitum access to food and water. Male and female mice were used for experiments in equal genotypic ratios. Female *Ube3a* ^*m+/p−*^ × male *Ube3a* ^*m+/p+*^ breeders generated littermate experimental *Ube3a* ^*m−/p+*^ (AS) and *Ube3a* ^*m+/p+*^ wild-type (WT) mice. We maintained separate 129 and C57BL/6 colonies, each congenic for 10+ generations. Ype Elgersma (Erasmus Medical Center) provided the 129 mice, and Jackson Labs (Bar Harbor, ME) provided the C57BL/6 mice (JAX #: 016590).

#### Surgeries and LFP recordings

Surgery and local field potential (LFP) recordings were conducted as previously described [30], with only minor modifications. We anesthetized adult mice and implanted tungsten microelectrodes (FHC) bilaterally in layer 4 of the primary visual cortex (coordinates relative to lambda, in mm, 0 A/P, +3.2–3.3 M/L, −0.47 D/V). We implanted a silver ground wire in the cerebellum and head-fixed mice using a steel headpost attached to the skull anterior to the bregma. Electrodes and headposts were held in place by headcaps made from dental cement (Metabond). Following surgeries, mice recovered for at least 2 days prior to 2 days of habituation (15 min) to the recording apparatus. We then recorded LFP continuously for 15 min on three consecutive days following habituation. Mice were head-fixed during all recordings and viewed a static gray screen in an otherwise dark, quiet environment. We amplified data 1000× using single-channel amplifiers (Grass Technologies), digitized data using a Micro 1401 digitizer (CED), and acquired data at 4 kHz using Spike2 software (CED). We applied analog 0.1-Hz high-pass and 100-Hz low-pass filtration during data acquisition and digital 1-Hz high-pass filtration (second-order Butterworth) after data acquisition. The roll-off of the high-pass Butterworth filter did not impinge onto the delta range of interest (2–4 Hz). Ages of mice on the first day of LFP recording ranged from P85 to P114 and averaged 94.0 ± 1.2 days (129) and 100.0 ± 0.8 days (C57).

#### LFP analysis

Sample size (“*n*”) represents the number of mice. For each mouse, we averaged processed data from the left and right hemispheres within a session, then averaged results across three sessions. For sessions with movement artifacts, we selected the longest continuous period with no artifacts present for analysis. We analyzed spectral power using a fast Fourier transform (FFT) of the continuous signal, resulting in frequency bins of 0.5 Hz. We determined relative power by expressing power in a given frequency band as a percentage of the total power between 1 and 50 Hz. A disadvantage of using relative power is that by definition, total power must summate to 100%, so a genotype difference in one frequency band (e.g., delta) may also manifest as relative genotype differences in other frequency bands (see “Results”). Thus, it is difficult to appropriately assess differences in relative power in frequency bands other than delta (Additional file 2: Figure S2). We defined delta as 2–4 Hz, theta as 5–10 Hz, beta as 13–30 Hz, and gamma as 30–50 Hz.

For each LFP electrode, we assessed delta dynamics (Fig. 2) by quantifying the spread of delta power in all 2-s bins with a 1-s overlap. We generated box plots of raw and relative delta power using 2-s bins for the duration of each recording and quantified three parameters: mean, interquartile range (IQR), and outliers, defined as Q1 − 1.5*IQR or Q3 + 1.5*IQR. Mean delta power calculated by averaging bins was statistically indistinguishable from delta power calculated by FFT on the entire signal (mean of bins—WT, 20.0 ± 2.2%; AS, 29.0 ± 2.4%; FFT of the entire signal—WT, 20.3 ± 2.0%; AS, 28.9 ± 2.3%, represented in Fig. 1j). IQR represents the spread between the middle 50% of delta measurements, and we used this as a readout of within-session delta dynamics. Suprathreshold outliers represent bouts of “strong delta.” We wrote custom MATLAB scripts to analyze delta dynamics and used Spike2 software for basic spectral analyses.

### Human EEG methods

#### Data sources

All EEG studies and analyses were performed with institutional review board (IRB) approval. We analyzed EEGs from 28 children with AS (14 males, 14 females) and 72 neurotypical controls (42 males, 30 females). During EEGs, 26/28 AS individuals had periods of wake and 13/28 had periods of sleep. During EEGs, 54/72 neurotypical individuals had periods of wake and 54/72 had periods of sleep. These samples (Table 1) represent the cross-sectional data analyzed in Fig. 3 (wake), Fig. 4 (sleep), Additional file 3: Figure S3, and Fig. 5a, b, e, f. For longitudinal studies, we analyzed repeat EEGs from 12 AS individuals, resulting in a total of 45 wake EEG sessions and 15 sleep EEG sessions (Fig. 5c, d).

**Table 1:** Characteristics of study subjects

	Neurotypical	Angelman
Total patients	72	28
Age (years)	7.0 ± 0.2	5.8 ± 0.3
Male	42 (58%)	14 (50%)
Female	30 (42%)	14 (50%)
Molecular diagnosis	N/A	Class 1 deletion: 7 Class 2 deletion: 10 UBE3A mutation: 6 Atypical deletion: 2 Uniparental disomy: 1 Imprinting defect: 1 Abnormal DNA methylation, negative for deletion: 1
History of seizures	0 (0%)	26 (93%)
Patients with wake in EEG	54 (75%)	26 (93%)
Age (years)	6.6 ± 0.3	5.8 ± 0.3
Male	30 (56%)	14 (54%)
Female	24 (44%)	12 (46%)
Wakeful EEG length (min)	7.9 ± 1.0	18.2 ± 2.3
Seizures under control at time of first recording or no seizure history	54 (100%)	24 (92%)
Patients with sleep in EEG	54 (75%)	13 (46%)
Age (years)	7.1 ± 0.3	6.0 ± 0.4
Male	32 (59%)	8 (62%)
Female	22 (41%)	5 (38%)
Sleep EEG length (min)	13.6 ± 0.8	22.0 ± 2.4
Seizures under control at time of first recording or no seizure history	54 (100%)	12 (92%)

#### Data acquisition, processing, and analysis

Both neurotypical EEGs (MGH) and AS EEGs (RCHSD) were performed using the standard clinical method. All data were recorded using the standard 10-20 EEG system using a common physical reference on either Bio-Logic or Xltek systems. The location of the physical reference varied between sites; therefore, we re-referenced all data to linked ears ((A1 + A2) / 2). Neurotypical EEGs were recorded at 200, 250, 500, or 512 Hz, and AS EEGs were recorded with a sampling rate of 256 or 512 Hz.

We processed all raw data from both sites using the same pipeline, which included re-referencing to linked ear reference, filtering, manual inspection by a board-certified neurophysiologist (CJC, GMD), sleep/wake coding, artifact removal, and analysis. After re-referencing, data were broken into sleep (NREM) and wake epochs by an experienced clinical neurophysiologist (CJC, GMD, MD). Periods in which wake/sleep state was unclear were excluded, and periods of REM sleep were also excluded. Next, data were digitally filtered (second-order Butterworth): 1-Hz high-pass, 100-Hz low-pass, and 60-Hz notch. Movement artifacts were manually marked and excluded. We used EEGLAB [31] as a viewer to assess sleep state and identify artifacts. Observers were not blind to genotype during EEG inspection, sleep/wake coding, and artifact removal.

After processing, we used custom MATLAB scripts to analyze all data. Neurotypical and AS EEGs were batch-processed using the same programs at the same time. We slightly modified scripts from mouse LFP analysis for human EEG analysis. For each of 19 recording electrodes, we generated power spectra and calculated delta power and delta dynamics. We group-averaged results from neighboring electrodes to assess delta phenotypes by region (Fig. 3a): occipital (O1, O2), temporal (T3, T4, T5, T6), parietal (P3, Pz, P4), central (C3, Cz, C4), and frontal (Fp1, Fp2, F3, Fz, F4, F8). We quantified relative power in all human data analyses to account for variability in the amplitude of raw signals (higher variability than seen in mouse). Sleep/wake coding and artifact removal resulted in noncontinuous signals. We did not concatenate processed signals together; instead, we analyzed all 2-s bins (with 1-s overlap) of active signal. We averaged spectra and delta power from all active bins. This approach diverged slightly from mouse LFP analysis, where we performed spectral analysis on the continuous signal. However, as noted above, adapting these methods to mice resulted in no change in the values of delta.

### Statistical analysis

In mouse, we compared power (raw or relative, as noted) in a given band of interest (delta, gamma, etc.) using Student’s *t* tests (Fig. 1e–i, Additional files 1: Figure S1C, D and 2: Figure S2D–J). We compared group delta distributions using a Kolmogorov-Smirnov (K-S) test (Fig. 2f). We assessed delta dynamics (IQR) and the number of strong delta bouts using Student’s *t* tests (Fig. 2g–i). In human, we compared delta power and delta dynamics (IQR) within each region using Student’s *t* tests (Figs. 3f, g and 4f, g). We assessed the effects of age and genotype on delta power and dynamics in a cross-sectional sample using a two-way ANOVA with age (as a continuous measure) and genotype as factors (Fig. 5a, b, e, f). As there was a significant main effect of age on delta power in the total sample, we used a post hoc one-way ANOVA with age (as a continuous measure) as a factor to the age dependence of delta power within the AS group (Fig. 5a). We assessed the effect of age on delta in a longitudinal sample using a linear mixed model examining the fixed main effect of age on either power or dynamics, including the random effect of age nested in each subject in order to account for individual differences in the ages and age intervals of each repeated measure (Fig 5c, d). We assessed the effects of age and genotype in a combined sample containing all EEG sessions using a two-way ANOVA with age and genotype as factors; we used Bonferroni tests to make post hoc comparisons between groups within each age range (Fig. 5g, h). We used GraphPad Prism and JMP software (SAS) to perform statistical analyses.

## Results

### Angelman syndrome model mice have increased delta power

We previously showed that deletion of *Ube3a* from GABAergic neurons, but not glutamatergic neurons, increased delta rhythmicity and caused an exaggerated increase in seizure susceptibility compared to AS model mice with pan-cellular loss of the maternal *Ube3a* allele (*Ube3a* ^*m−/p+*^) [30]. While these studies provided insights into the importance of *Ube3a* loss in GABAergic neurons to hyperexcitability phenotypes in AS, it is critically important to fully assess whether *Ube3a* ^*m−/p+*^ mice accurately reflect clinical EEG phenotypes and to establish objective measures in both the preclinical mouse model and the clinical AS population. Towards this goal, we first quantified delta in *Ube3a* ^*m−/p+*^ mice and wild-type littermate controls (*Ube3a* ^*m+/p+*^). Because delta rhythmicity was reported to be strong in the occipital cortex in humans with AS [20, 21], we implanted electrodes into layer 4 of the primary visual cortex and recorded local field potentials (LFPs) (Fig. 1a). Direct brain implantation distinguishes LFP recordings from traditional scalp EEG and provides a more accurate reflection of local neural activity [32]. To approximate a resting state, we recorded LFP in awake, head-fixed mice viewing a static gray screen in a dark, quiet environment to which they were previously habituated. We compared AS model mice to wild-type littermates separately in two commonly used mouse strains in AS research: 129 and C57BL/6.

AS model mice on a 129 background showed enhanced delta (2–4 Hz) power (Fig. 1b–e). Genotypic differences in LFP power were restricted to the delta band (Fig. 1f–i). LFP power within a band of interest is often represented as a fraction of total power (relative power), and we found that relative delta power was also significantly increased in AS model mice (Fig. 1j). However, for other frequency bands, genotypic differences in relative power must be interpreted with caution: because total power must summate to 100%, increases in delta (which normally accounts for a disproportionate ~20% of total power) may also manifest in artifactual or misleading relative power differences in other bands. For example, AS model mice displayed statistically decreased relative theta and relative gamma power (Additional file 1: Figure S1), despite normal raw power in these bands. Therefore, raw and relative analyses of delta power may be interchangeable in AS model mice, but group differences in relative power outside of delta can be misleading if delta itself shows group differences.

Delta power in the primary visual cortex was not significantly different between WT and AS mice on a C57BL/6 background (Additional file 2: Figure S2A–F). AS mice showed a trend towards increased raw power in the 3–5 Hz range (Additional file 2: Figure S2G) and a statistically significant increase in relative 3–5 Hz power (Additional file 2: Figure S2H). Total power (1–50 Hz) was not different as a function of genotype (Additional file 2: Figure S2I). Surprisingly, gamma power (both raw and relative) was decreased in AS mice on a C57 background (Additional file 2: Figure S2J, K). Beta power (both raw and relative) were not different as a function of genotype (Additional file 2: Figure S2L, M).

### Angelman syndrome model mice exhibit more dynamic delta oscillations

We sought to understand the nature of increased delta power in AS model mice. Broadly, the overall increase in delta power in AS could be driven by (a) short bouts of very strong delta, (b) a consistent moderate increase in delta, or (c) a more complex pattern. We thus quantified the distribution of delta power across time within individual recordings. First, we quantified delta power during every 2-s window of continuous LFP recordings (Fig. 2a, b) and analyzed the distribution of these measurements (Fig. 2c, d). On both 129 and C57 backgrounds, WT and AS mice had statistically different distributions of delta power over time, with AS distributions shifted towards having more periods of stronger delta. However, this approach—group averaging of individual delta distributions—is unable to determine whether group differences between WT and AS are driven by within-animal differences or across-animal differences. Therefore, we assessed delta variability, or dynamics, within single recording sessions. Within each session, we represented delta power in every 2-s window as a box plot. We quantified delta dynamics in two ways: (1) interquartile range (IQR), as a proxy for the range of “typical” delta, and (2) fraction of suprathreshold bins (where threshold = Q3 + 1.5*IQR), as a way to assess the amount of “strong” delta bouts (Fig. 2e, f). AS mice showed increased IQR, indicating that delta power is more dynamic within a session. Delta was more dynamic in AS mice on both 129 and C57 backgrounds, using both raw and relative power as measures (Fig. 2e, f). There was no increase in the number of strong delta bouts in AS mice on either background (Fig. 2g), indicating that delta power phenotypes in AS model mice were not driven by discrete bouts of abnormally strong delta oscillations. There were actually fewer strong delta bouts in AS mice relative to WT, but this was likely driven by increased IQR in these mice, resulting in a higher threshold for defining a strong bout. Overall, we found that delta power was more variable across time (i.e., “dynamic”) within single recording sessions in AS model mice.

### Children with Angelman syndrome exhibit enhanced delta power and dynamics

Employing similar methods used to quantify mouse LFPs, we compared delta power and dynamics from retrospective clinical EEGs in children with AS and age-matched neurotypical controls. EEG recordings contained periods of both wakefulness and sleep, presenting a potential confound. Children with AS have severe sleep disturbances [1], potentially biasing their EEG recordings towards wakefulness. Indeed, 15/47 total EEGs from AS individuals included periods of sleep (32%), while 54/72 total EEGs from neurotypical individuals included periods of sleep (75%). Because enhanced delta is a signature of slow-wave sleep [33], we separately analyzed EEG in wake and sleep states between groups. Another potential confound was the high incidence of epilepsy (80-95%) in AS patient populations [1]. In our sample, 26/28 AS individuals (93%) had a history of seizures (1 no seizures, 1 unknown), and no neurotypical individuals had a history of seizures (Table 1; for raw data, see Additional file 4). However, most (24/26) children with a history of seizures were on at least one medication at the time of their first EEG, and most (24/26) children’s seizures were under control at the time of their first EEG (1 with persistent seizures, 1 unknown).

We quantified delta power and dynamics for each EEG electrode and group-averaged neighboring electrodes by region (Fig. 3a). During wakefulness, children with AS (*n* = 26) showed strongly increased delta power and delta dynamics relative to neurotypical controls (*n* = 54) (Fig. 3b–g; Additional files 3: Figure S3 and 5: Figure S4A–C). Delta power and dynamics were increased in every spatially defined region, suggesting that delta phenotypes generalize across the neocortex. While delta phenotypes were present across all recording areas in group-averaged data, individual recordings did show some spatially restricted delta bouts (Additional file 5: Figure S4D–F). As expected, periods of manually identified sleep showed increased delta power relative to periods of wakefulness in both AS and neurotypical children (Fig. 4a; compare Figs. 3f and 4f). During sleep, delta power and dynamics were increased in children with AS (*n* = 13) relative to neurotypical controls (*n* = 54) in all regions (Fig. 4b–g; Additional file 3: Figure S3). Manual inspection of traces revealed that our sample included other EEG signatures, such as “notched” delta (Additional file 5: Figure S4G–I), that have been previously reported in children with AS [20]. As some antiepileptic medications are known to cause EEG slowing [34], we confirmed that the two children with AS not taking medication displayed elevated delta power (awake occipital relative delta power in NT, 21.7 ± 0.6%; in AS, 39.3 ± 1.6%; in child 1, age 4, 49.6%; in child 2, age 5, 52.1%). Thus, it is not likely that delta phenotypes in children with AS were caused by antiepileptic medications.

### Delta power in Angelman syndrome is age-dependent

Our initial sample (analyzed in Figs. 3 and 4) included one EEG session per child, age 4–11. This cross-sectional sample showed an age-dependent decrease in occipital delta power during wakefulness, independent of genotype (Fig. 5a; two-way ANOVA, main effect of genotype: *p* < 0.0001, main effect of age: *p* = 0.0011). Occipital delta power decreased with age in children with AS (*p* = 0.041, post hoc test); this result supports qualitative clinical observations from a sample of children with AS ranging in age from 0.4 to 25 years [21]. However, there was no statistical difference in delta power trajectories between AS and neurotypical groups (genotype × age interaction: *p* = 0.0801). Occipital delta dynamics during wakefulness (Fig. 5b) also varied with genotype (*p* < 0.001), though there was not a statistically significant effect of age on dynamics (*p* = 0.069) or an interaction between genotype and age (*p* = 0.769).

If delta phenotypes are to be a useful biomarker in AS, they must remain stable or follow a predictable developmental trajectory within subjects. We thus quantified delta power and dynamics longitudinally in a subset of AS individuals from the original sample, where follow-up EEG recordings were available. We analyzed longitudinal EEGs (two to four per child) from 13/28 children, spanning up to 7 years. Twelve of 13 children had multiple recordings with periods of wakefulness; only two of 13 had multiple recordings with periods of sleep. Within subjects, there was a significant main effect of age on delta power during wakefulness (*p* < 0.0001), confirming that individuals showed developmental trajectories of reduced delta in line with cross-sectional data (Fig. 5c). Longitudinal assessment also revealed a significant main effect of age on delta dynamics (IQR) in children with AS (*p* = 0.0003; Fig. 5d). During sleep, cross-sectional analyses revealed that delta power in AS individuals was not significantly age-dependent (Fig. 5e; two-way ANOVA, main effect of genotype: *p* < 0.0001, main effect of age: *p* = 0.458, genotype × age interaction: *p* = 0.658). Additionally, delta dynamics during sleep were not significantly age-dependent (Fig. 5f; main effect of genotype: *p* < 0.0001, main effect of age: *p* = 0.259, genotype × age interaction: *p* = 0.645).

Overall, cross-sectional and longitudinal analyses indicated that during wakefulness, delta phenotypes in AS were more pronounced at earlier ages. We next sought to determine whether enhanced delta rhythms persisted in older children despite the developmental trajectory in AS individuals towards reduced delta. Overall, we compared 44 awake EEGs from 26 children with AS (combined cross-sectional and longitudinal data) to 54 wake EEGs, one per neurotypical child, and assessed delta phenotypes in three age ranges (in years): 4–6, 6–8, and 8+. During wakefulness, delta power and delta dynamics were significantly increased at all ages in children with AS (Fig. 5g). During sleep (AS: *n* = 15 sessions from 13 children; NT: *n* = 54 sessions, one per child), delta power and dynamics were significantly increased at age 4–6 and age 6–8; we analyzed only one sleep EEG from a child with AS older than 8 (Fig. 5h).

## Discussion

Rhythmic delta is the most pervasive EEG abnormality in AS, but delta phenotypes have not been previously quantified. If delta oscillations are to be an effective biomarker, quantitative methods are required to track acute or longitudinal changes in rhythmicity. Here, we used spectral analyses to confirm that delta abnormalities in AS model mice mirror clinical reports from the AS patient population (Fig. 1). Using similar methods, we quantified robust delta phenotypes in children with AS across the neocortex during wake and sleep (Figs. 3 and 4), showing that the enhanced delta phenotype scales in a state-dependent manner. The enhanced delta activity in AS individuals followed a predictable developmental trajectory across subjects and within subjects (Fig. 5). While delta phenotypes were stronger at earlier ages, they persisted in all age groups tested (4–11 years), demonstrating that delta activity may be useful as a longitudinal biomarker, in addition to its utility as an acute biomarker in young children. Spectral analyses revealed increased dynamics, or variability, of delta oscillations within single sessions in both AS model mice and children with AS (Figs. 2 and 4). This phenotype had not been described in a patient population and would be difficult to visualize and assess clinically without quantitative methods.

With multiple approaches currently being developed for clinical trials in AS, reliable and robust biomarkers are needed. Characteristics of a strong disease biomarker also include face validity and evidence for reversibility in a mouse model. Here, we showed that abnormal delta rhythmicity is conserved between mouse models and patient populations in AS, and prior work showed that increased delta power may be reversed in AS model mice by embryonic reinstatement of the UBE3A protein in a subset of neurons [30]. To date, phenotypic behaviors have been characterized in AS model mice with varying reliability [35] and include sensory, motor, and learning impairments [11, 23]. Taken together, mouse behavioral phenotypes generally resemble human symptoms, but their direct face validity is limited and, thus, are not ideal biomarkers. One exception to this rule is seizures, which may be robustly and reliably induced in AS mouse models [23, 30]. However, the use of seizures as a biomarker in AS children is limited; seizures are typically treated with antiepileptic medications and are controlled to a great extent in the majority of children [36]. Delta rhythmicity represents a robust, reliable biomarker with strong face validity between mouse models and patient populations.

We observed strain differences in delta phenotypes in AS model mice: delta power (2–4 Hz) was increased in AS on a 129 background (Fig. 1), but not on a C57BL/6 background (Additional file 2: Figure S2), despite a trend towards increased power in the 3–5-Hz band. Despite statistically normal delta power, AS model mice on a C57BL/6 background did show increased delta dynamics (Fig. 2). Thus, while delta power phenotypes may be strain-specific, abnormal delta dynamics are preserved across two commonly used strains for AS research. Strain differences in delta power are not surprising, as behavioral differences have also been noted between AS mice on 129 and C57BL/6 backgrounds [35].

Quantitative assessment of retrospective human EEG data revealed a robust increase in delta power in children with AS. These results support clinical reports [14–22], and our data validate the utility of quantifying delta activity pre- and post-intervention to track acute and sustained consequences of therapeutic interventions. Spectral analyses also address the nature of delta abnormalities in AS in a manner not possible by clinician review. Our study of within-session delta dynamics revealed that delta oscillations are more variable in AS, but are not confined to intermittent bouts.

Clinically, delta abnormalities have been observed in both posterior (73% of patients) and anterior (59%) regions [21], with potential differences in the type of delta seen by region [37]. We found that delta phenotypes (increased power and dynamics) generalized across the neocortex in a large sample (Figs. 3 and 4). However, spatially restricted runs of delta were observed within individual recordings (Additional file 5: Figure S4). Additionally, while spectral analyses provide an unbiased method to quantify power within a band of interest, a disadvantage of their use is an inability to dissociate subtle variants of delta, such as notched delta (Additional file 5: Figure S4), which have been noted in clinical studies of AS [17, 20, 22]. Thus, spectral analyses are best suited for quantifying broad delta biomarkers. We chose to focus on delta rhythmicity because it is the most common EEG abnormality in AS and the most specific abnormality to AS relative to related disorders [20, 24]. However, interictal epileptiform discharges and theta abnormalities have also been widely reported [14, 17, 20, 21, 38]. Epileptiform discharges are typically coincident with rhythmic delta [37] and are therefore likely captured by using delta power as a biomarker; our analyses did not distinguish epileptiform discharges in the 2–4-Hz frequency band from background delta rhythms. Increased theta (~4–6 Hz in human) has been noted in ~30–60% of children with AS (Additional file 5: Figure S4), but is age-dependent and rarely observed beyond age 8 [17, 20, 21]. Thus, we were not surprised to see normal theta in adult AS model mice (Fig. 1, Additional file 2: Figure S2). Quantitative assessment of theta and other bands in human EEG data were complicated by our a priori hypothesis that delta is increased and by the limits imposed by quantifying relative power (see “Results” or “Methods”).

Enhanced delta rhythmicity is a signature of slow-wave sleep, and our quantification confirmed that delta rhythms are indeed increased in neurotypical individuals during sleep epochs (Fig. 4). In AS individuals, delta rhythms are also increased during sleep relative to wakefulness, and thus, the enhanced delta phenotypes are preserved and scaled with state changes. These data show that it is critical to identify and separate wakeful and sleep epochs during EEG recordings but that delta remains an effective biomarker when making state-specific comparisons. Enhanced delta does not appear to broadly disrupt sleep architecture. Children with AS show typical sleep architecture such as sleep spindles and vertex waves. While there may be some disruption in sleep architecture, these appear to be minor compared to the significant effects of sleep-activated discharges on sleep architecture.

In addition to generalizing across sleep and wake, delta phenotypes in AS are also present across childhood development. We found a developmental reduction in delta power in AS; however, delta phenotypes persisted in all age ranges tested, to 12 years (Fig. 5). Thus, delta remains a valid biomarker throughout childhood and may be used as interventions and clinical trials are likely to occur in children of all ages. It is not clear whether the developmental attenuation of delta phenotypes is directly linked to loss of UBE3A. The attenuation of delta activity may be related to a secondary feature of AS, such as improvements in epilepsy and sleep at older ages [39]. It is also not yet known how delta phenotypes correlate with clinical features of AS such as epilepsy severity, sleep, and behavioral, cognitive, and motor impairments. However, in mice, cell type-specific manipulations of UBE3A that increase delta power also increase seizure susceptibility, and those that do not affect delta also do not affect seizures [30].

Our work represents the first direct comparison of EEGs from children with AS and neurotypical controls. However, an inherent limit of our retrospective EEG analyses was that AS data and neurotypical data were gathered at two different sites. We processed and analyzed all data in parallel and were encouraged by the robustness of phenotypes, but future prospective studies should be designed to recruit AS and control patients to a single site. In addition, intellectual disability in children with AS presents a potential confound, as EEG slowing has been associated with cognitive impairment in several populations [40, 41]. Future work comparing AS to other reference groups (i.e., nonsyndromic seizure, intellectual disability, autism) will be critical to understanding the extent to which other disorders may exhibit delta phenotypes. AS may be considered an autism-like disorder, as a subset of children with AS also meet the diagnostic criteria for autism [42–44]. Quantitative EEG methods have characterized some spectral and coherence phenotypes in nonsyndromic autism [29, 45–51], yet the genetic heterogeneity of nonsyndromic autism introduces challenges in finding common EEG biomarkers. However, recent work has identified EEG signatures of Dup15q syndrome, a syndromic form of autism caused by duplication of the 15q11-13 genetic region which includes *UBE3A* [4–6]. The most profound EEG abnormality in Dup15q is increased beta rhythmicity, which is normal in AS model mice (Fig. 1, Additional files 1: Figure S1 and 2: Figure S2), but decreased delta power has also been noted in Dup15q individuals [52–54]. Thus, bidirectional changes in *UBE3A* gene dosage are linked to mirror symmetric changes in delta power, suggesting a critical role for UBE3A protein in regulating delta-generating brain circuits.

Fragile X syndrome, another single-gene disorder associated with autism, provides a case study in the importance of defining reliable biomarkers for use as clinical outcome measures. A series of mechanism-based pharmacological studies in mice sought to normalize synaptic protein synthesis, a key pathological feature of Fragile X [55–57]. Pharmacological interventions directed towards normalizing protein synthesis were highly successful in correcting Fragile X phenotypes in mice [58–60], ultimately leading to multiple phase 2 clinical trials [61, 62]. These well-designed and well-powered trials ultimately failed because no improvements were seen in predefined behavioral endpoints [63]. While other aspects of these studies were also relevant to their outcomes, such as the age of children enrolled and the duration of treatments, this work provides a rationale to develop biologically based, quantitative, robust, and repeatable outcome measures for clinical trials. We propose that delta rhythmicity meets these criteria for Angelman syndrome.

## Conclusions

Delta rhythmicity phenotypes are quantifiable and robust in children with Angelman syndrome and in mouse models of the disorder. Delta phenotypes have strong face validity between mouse models and patient populations; thus, future mechanistic studies of delta rhythms in mice will have high translational potential. In patient populations, delta phenotypes have value as biomarkers to chart progression of AS and as clinical outcome measures.

## Additional files

Additional file 1: Figure S1. Quantifying relative power preserves delta phenotypes in AS model mice, but complicates interpretations in other bands. (A, B) Relative power in 129 mice (WT: *n* = 23, AS: *n* = 24), plotted as a fraction of total power (1–50 Hz). Quantification of relative (C) theta, (D) beta, and (E) gamma power. Relative theta and gamma are significantly decreased in AS model mice on a 129 background (theta: *p = 0.013, beta: *p* = .209, gamma: **p = 0.0007, Student’s *t* test). (PDF 131 kb)

Additional file 2: Figure S2. Strain differences in the primary visual cortex LFP power in Angelman syndrome model mice. (A, B) Power spectra of group data (WT: *n* = 30, AS: *n* = 39; shading indicates ±sem) from the primary visual cortex in C57BL/6 mice. (C, D) Power spectra, measured relative to total power. (E) Raw and (F) relative delta power are not different between WT and AS (raw: *p* = 0.277, relative: #*p* = 0.073, Student’s *t* tests). (G) Raw power in the 3–5-Hz band is not different between WT and AS (#*p* = 0.077). (H) Relative power in the 3–5-Hz band is significantly increased in AS model mice (**p = 0.0052). (I) Total power (1–50 Hz) is not different between groups (*p* = 0.460). (J) Raw and (K) relative gamma power are decreased in AS model mice on a C57BL/6 background (raw: *p = 0.022, relative: ***p = 0.00074). (L) Raw and (M) relative beta power are not different between groups (raw: *p* = .476, relative: *p* = .166) (PDF 179 kb)

Additional file 3: Figure S3. Power spectra from all regions during epochs of wake and sleep. Black: neurotypical (NT), red: AS. During wakefulness (NT: *n* = 54, AS: *n* = 26), (A) occipital, (B) temporal, (C) parietal, (D) central, and (E) frontal spectra. During sleep (NT: *n* = 54, AS: *n* = 13), (F) occipital, (G) temporal, (H) parietal, (I) central, and (J) frontal spectra. (PDF 896 kb)

Additional file 4: Seizure and medication history for children with AS. This file is a table that provides the following information for each child with AS where it was available: (1) age at EEG, (2) gender, (3) molecular diagnosis, (4) history of seizures (yes/no), (5) age of onset of seizures, (6) seizures controlled at the time of EEG (yes/no), (7) types of seizures in the past, and (8) medications at the time of EEG. (XLS 36 kb)

Additional file 5: Figure S4. Examples of EEG variants in children with Angelman syndrome. (A–C) Three examples of enhanced delta oscillations generalized across the neocortex. (D) An example of delta oscillations restricted to posterior electrodes. (E) An example of delta oscillations restricted to frontal electrodes. (F) An example of delta oscillations restricted to frontal electrodes over the left hemisphere. (G, H) Examples of notched delta. (I) An example of theta oscillations. (PDF 9020 kb)

## Abbreviations

AS: Angelman syndrome
EEG: Electroencephalography
IQR: Interquartile range
LFP: Local field potential
