# Erratum to: Delta rhythmicity is a reliable EEG biomarker in Angelman syndrome: a parallel mouse and human analysis

**DOI:** 10.1186/s11689-017-9210-0

**Published:** 2017-07-17

**Authors:** Michael S. Sidorov, Gina M. Deck, Marjan Dolatshahi, Ronald L. Thibert, Lynne M. Bird, Catherine J. Chu, Benjamin D. Philpot

**Affiliations:** 1 Department of Cell Biology and Physiology, University of North Carolina, Chapel Hill, NC 27599 USA; 2 Carolina Institute for Developmental Disabilities, University of North Carolina, Chapel Hill, NC 27599 USA; 3 Neuroscience Center, University of North Carolina, Chapel Hill, NC 27599 USA; 4 Department of Neurology, Massachusetts General Hospital, Boston, MA 02114 USA; 5 Harvard Medical School, Boston, MA 02215 USA; 6 Department of Pediatrics, University of California, San Diego, CA USA; 7 Division of Dysmorphology/Genetics, Rady Children’s Hospital, San Diego, CA USA; 8 Present Address: The Neurology Foundation, Rhode Island Hospital and Warren Alpert School of Medicine at Brown University, Providence, RI 02903 USA

## Erratum

After publication of our article [1], we became aware that there were two minor data loading and analysis scripting errors in the human EEG data processing pipeline. These errors affected the channel loading/grouping and sleep/wake coding of EEG data. We have re-analysed all the data affected by these errors. The errors do not affect any interpretations or conclusions, thus no changes to the text are required apart from correcting *p* values and raw values affected by the errors. There are no changes to statistical significance or lack-thereof. The errors affect data presented in Fig. 3, Fig. 4, Fig. 5, and Additional file 3: Figure S3 and thus we have re-plotted these figures (see below).

**Fig. 3:**
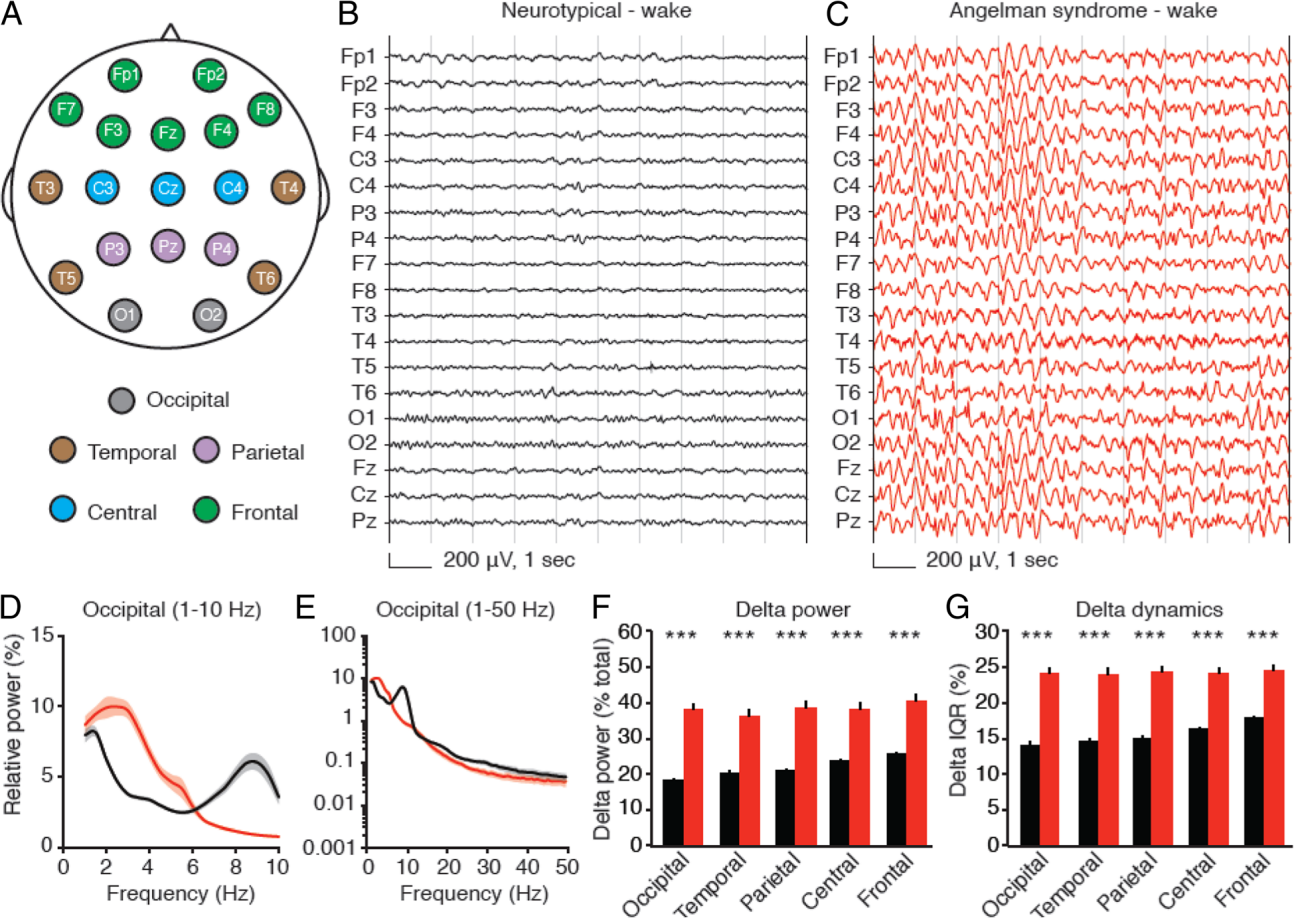
Delta rhythmicity is increased in children with Angelman syndrome relative to neurotypical controls during wakefulness. *Black*: neurotypical, *red*: AS. **a** Schematic showing EEG electrode placement according to the 10-20 recording system. Delta power and dynamics are calculated for each electrode and results averaged by region. Representative EEGs from **b** a neurotypical child and **c** a child with AS illustrate enhanced delta power, generalized across recording sites. **d**, **e** Power spectra of group data from occipital electrodes (NT: *n* = 54, AS: *n* = 26; *shading* indicates ± sem) illustrate an increase in delta power in AS; other regional spectra are shown in Additional file 3: Figure S3. **f** Group analyses reveal increased delta power generalizes across the neocortex (***p < 0.0001, Student’s *t* test). **g** Delta dynamics (IQR) are also increased in all regions (***p < 0.0001)

**Fig. 4:**
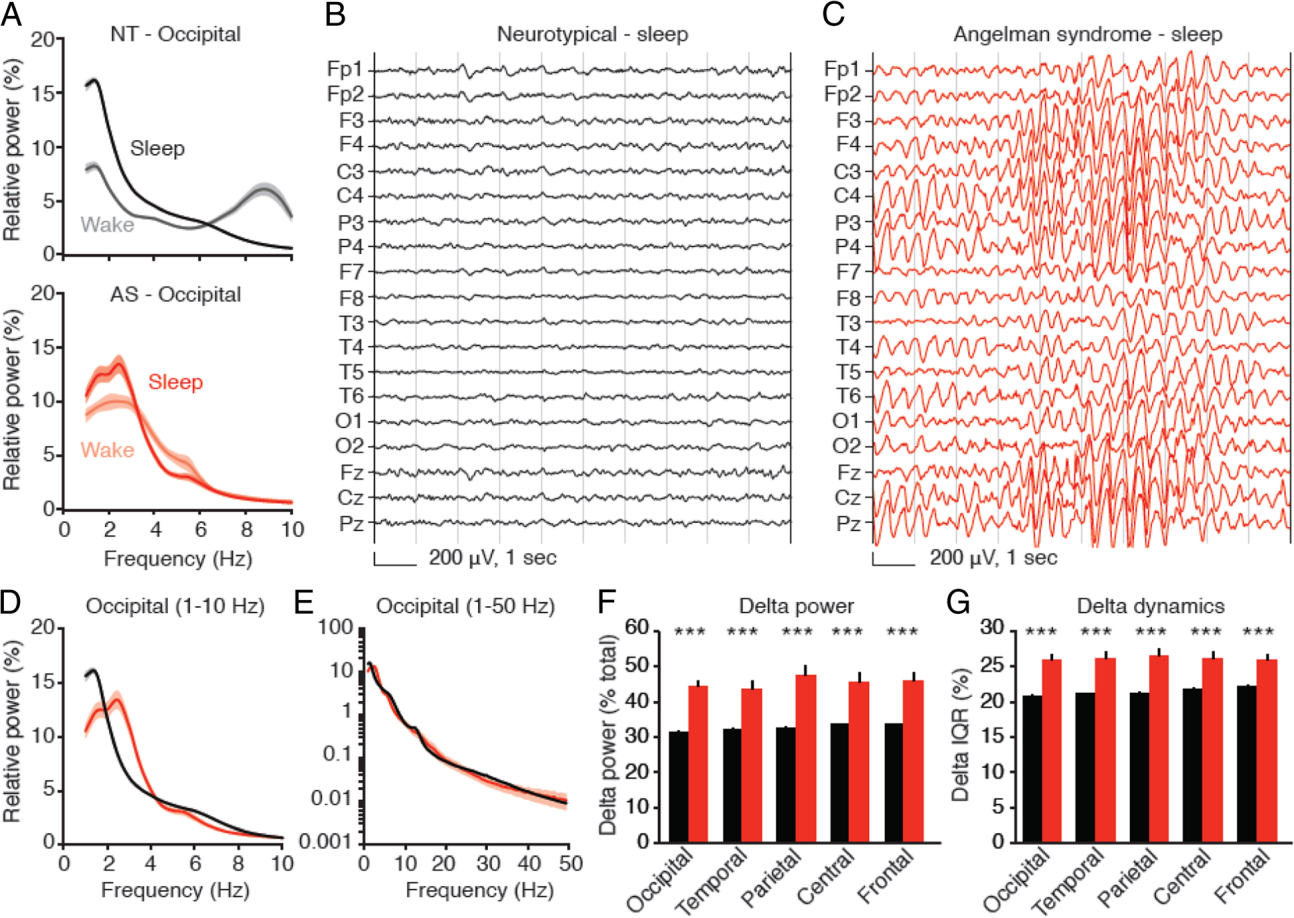
Delta rhythmicity is increased in children with Angelman syndrome relative to neurotypical controls during sleep. *Black*: neurotypical (NT), *red*: AS. **a** Occipital power spectra comparing wakefulness and sleep in neurotypical and AS children. Wake data are re-plotted from Fig. 3d; sleep data are re-plotted in **d**. Representative sleep EEGs from **b** a neurotypical child and **c** a child with Angelman syndrome illustrate delta oscillations in AS. **d**, **e** Occipital power spectra during sleep (NT: *n* = 54, AS: *n* = 13; *shading* indicates ± sem) show an increase in delta power in AS. **f** Group analyses reveal increased delta power generalizes across the neocortex (***p < 0.0001, Student’s t tests). **g** Delta dynamics (IQR) are also increased in all regions (***p < 0.0001)

**Fig. 5:**
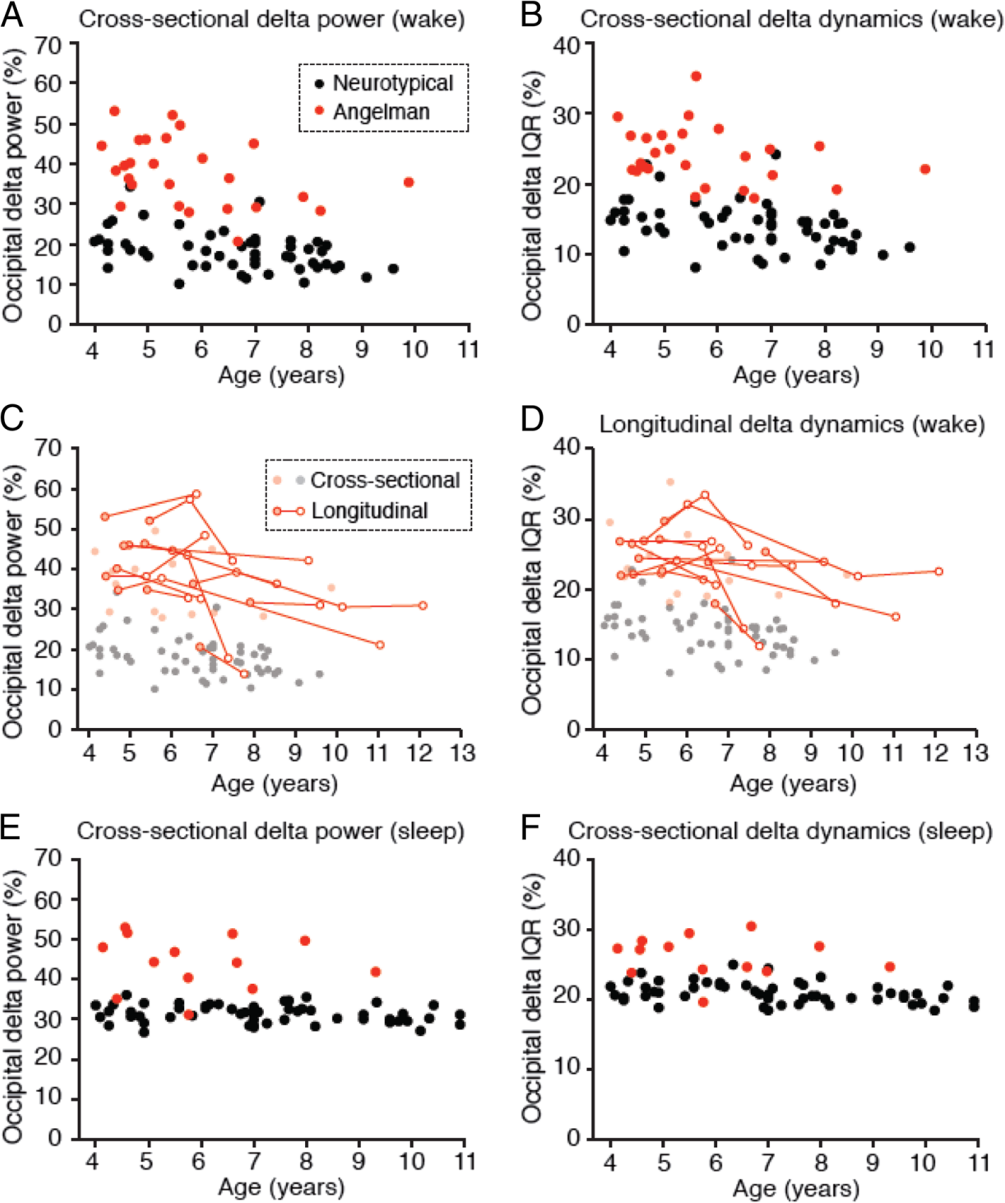
Delta phenotypes are stronger at earlier ages in children with Angelman syndrome. **a** Increased occipital delta power in children with AS is age-dependent during wakefulness (NT: *n* = 54, AS: *n* = 26). **b** Occipital delta dynamics as a function of age in neurotypical and AS children. Longitudinal studies in a subset of AS patients show that **c** delta power and **d** delta dynamics decrease as a function of age (*n* = 12 children, *n* = 31 sessions). **e** Delta power during sleep (NT: *n* = 54, AS: *n* = 13) and **f** delta dynamics during sleep do not show statistical age dependence. **g**, **h** Analysis of grouped cross-sectional and longitudinal occipital delta power and dynamics during wakefulness and sleep. **g** Delta power during wakefulness was increased in AS at ages 4–6, 6–8, and 8+ (two-way ANOVA and post hoc Bonferroni: ***p < 0.0001). Delta dynamics (IQR) during wakefulness were increased in AS at ages 4–6, 6–8, and 8+ (***p < 0.0001). Sample sizes are represented in bars. **h** Delta power and dynamics during sleep were increased in AS at ages 4–6 and 6–8 (***p < 0.0001)

More information on the errors:

The first error was in the script used to load and pre-process a subset of neurotypical EEG files. This error affected only neurotypical EEG files, as their raw formatting was different from Angelman syndome (AS) EEG files. The error in the loading script resulted in nine channels being mislabelled. The erroneous channel mapping was as follows:

- Reported channel/Actual channel
  - O1/Fpz*
  - O2/O1
  - P3/O2*
  - P4/P3
  - Pz/P4
  - T3/Pz*
  - T4/T3
  - T5/T4
  - T6/T5

For analysis, we averaged all data by region (i.e. O1 and O2 = occipital). Therefore only the three channels noted above with an asterisk were loaded in a way that impacted data analysis. This error affected a subset of neurotypical data presented in Fig. 3, Fig. 4, Fig. 5, and Additional file 3: Figure S3. We have corrected these figures, re-run all statistical tests, and corrected *p* values as detailed below.

In Table 1 the wakeful EEG length of 18.2 ± 2.3 min for children with Angelman syndrome should be replaced by 15.1 ± 2.3 min.

In the subsection “Children with Angelman syndrome exhibit enhanced delta power and dynamics” the last sentence should read: “As some antiepileptic medications are known to cause EEG slowing [34], we confirmed that the two children with AS not taking medication displayed elevated delta power (awake occipital relative delta power in NT, 18.2 ± 0.7%, in AS 37.9 ± 1.6%; in child 1, age 4, 49.5%; in child 2, age 5, 53.1%.”

In the first paragraph of the subsection “Delta power in Angelman syndrome is age-dependent” *p* = 0.0011 should read *p* = 0.0003, *p* = 0.041 should read *p* = 0.044, *p* = 0.0801 should read *p* = 0.2862, *p* = 0.069 should read *p* = 0.052, and *p* =0.769 should read *p* = 0.962.

In the second paragraph of the same subsection, *p* = 0.0003 should read *p* = 0.0009, *p* =0.458 should read *p* = 0.356, *p* = 0.658 should read *p* =0.775, *p* = 0.259 should read *p* =0.188, and *p* =0.645 should read *p* = 0.894.

In the figure legend for Fig. 5, *p* = 0.0002 should read *p* < 0.0001, and *p* = 0.0007 should read *p* < 0.0001.

The second error was in reading files containing sleep/wake annotations. These files contained time stamps followed by a code indicating “sleep”, “wake”, or “drowsy/unsure”. All sleep data were loaded correctly and were not affected by this error. The error occurred in a subset of wake data. A subset of EEGs that we reported as “awake” also included short periods of “drowsy/unsure.” Correcting this error resulted in tiny adjustments to the values of delta and the power spectra reported. These adjustments may not be visible beyond perhaps a “jitter” of a few AS data points in Fig. 5a–d. A smaller subset (3) of EEGs that we reported as “awake” did include periods of defined sleep, and this has now been corrected.

The corrected figures are as follows:

## Additional file

Additional file 3: Figure S3. Power spectra from all regions during epochs of wake and sleep. *Black*: neurotypical (NT), *red*: AS. During wakefulness (NT: *n* = 54, AS: *n* = 26), **a** occipital, **b** temporal, **c** parietal, **d** central, and **e** frontal spectra. During sleep (NT: *n* = 54, AS: *n* = 13), **f** occipital, **g** temporal, **h** parietal, **i** central, and **j** frontal spectra. (DOC 138 kb)
