# Supplementary figures and images for: Delta rhythmicity is a reliable EEG biomarker in Angelman syndrome: a parallel mouse and human analysis
Source: J Neurodev Disord. 2017 May 8;9:17. doi: 10.1186/s11689-017-9195-8 (PMC5422949; doi:10.1186/s11689-017-9195-8)

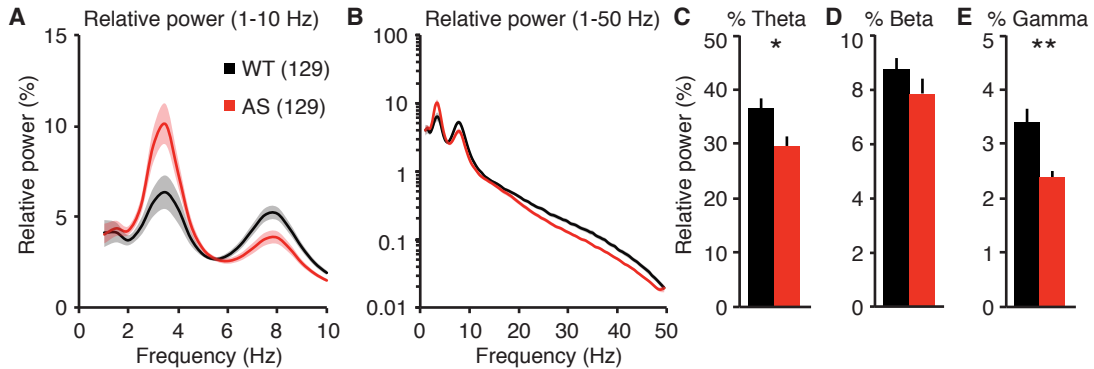

Supplement: Supplementary file 1 — Quantifying relative power preserves delta phenotypes in AS model mice, but complicates interpretations in other bands. (A, B) Relative power in 129 mice (WT: n = 23, AS: n = 24), plotted as a fraction of total power (1–50 Hz). Quantification of relative (C) theta, (D) beta, and (E) gamma power. Relative theta and gamma are significantly decreased in AS model mice on a 129 background (theta: *p = 0.013, beta: p = .209, gamma: **p = 0.0007, Student’s t test). (PDF 131 kb) [file 11689_2017_9195_MOESM1_ESM.pdf]

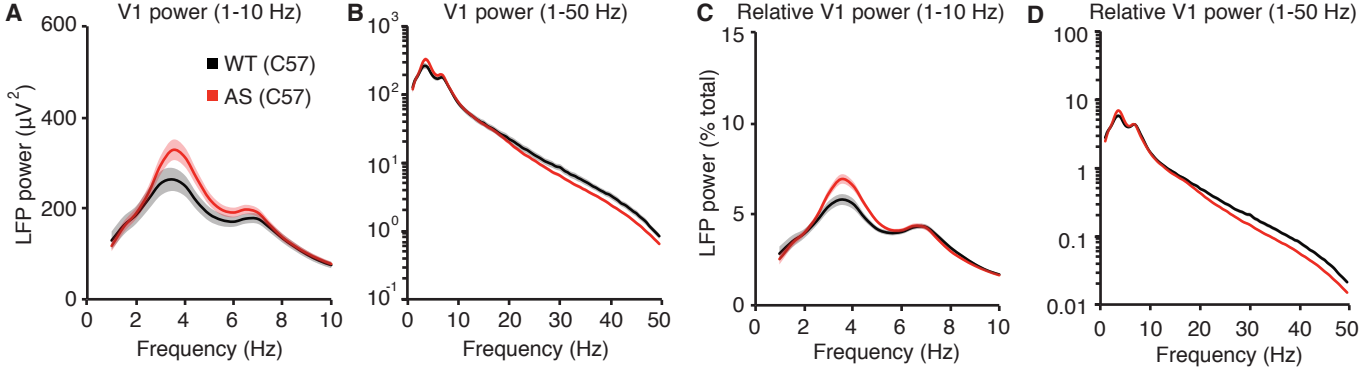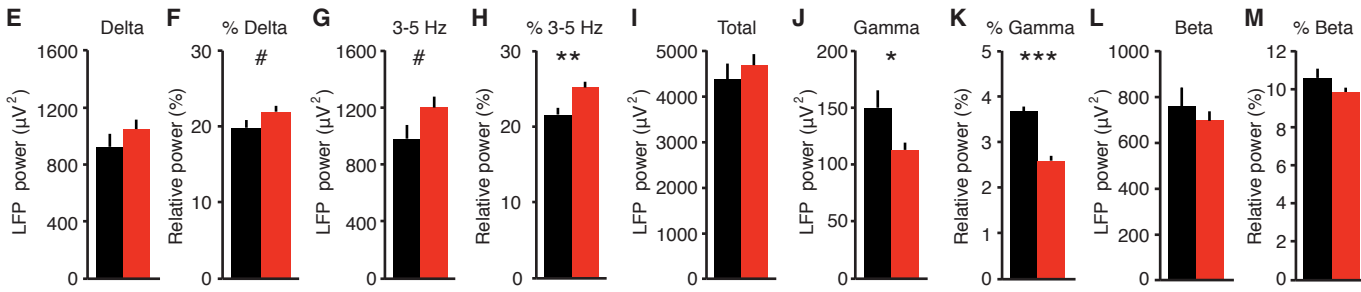

Supplement: Supplementary file 2 — Strain differences in the primary visual cortex LFP power in Angelman syndrome model mice. (A, B) Power spectra of group data (WT: n = 30, AS: n = 39; shading indicates ±sem) from the primary visual cortex in C57BL/6 mice. (C, D) Power spectra, measured relative to total power. (E) Raw and (F) relative delta power are not different between WT and AS (raw: p = 0.277, relative: #p = 0.073, Student’s t tests). (G) Raw power in the 3–5-Hz band is not different between WT and AS (#p = 0.077). (H) Relative power in the 3–5-Hz band is significantly increased in AS model mice (**p = 0.0052). (I) Total power (1–50 Hz) is not different between groups (p = 0.460). (J) Raw and (K) relative gamma power are decreased in AS model mice on a C57BL/6 background (raw: *p = 0.022, relative: ***p = 0.00074). (L) Raw and (M) relative beta power are not different between groups (raw: p = .476, relative: p = .166) (PDF 179 kb) [file 11689_2017_9195_MOESM2_ESM.pdf]

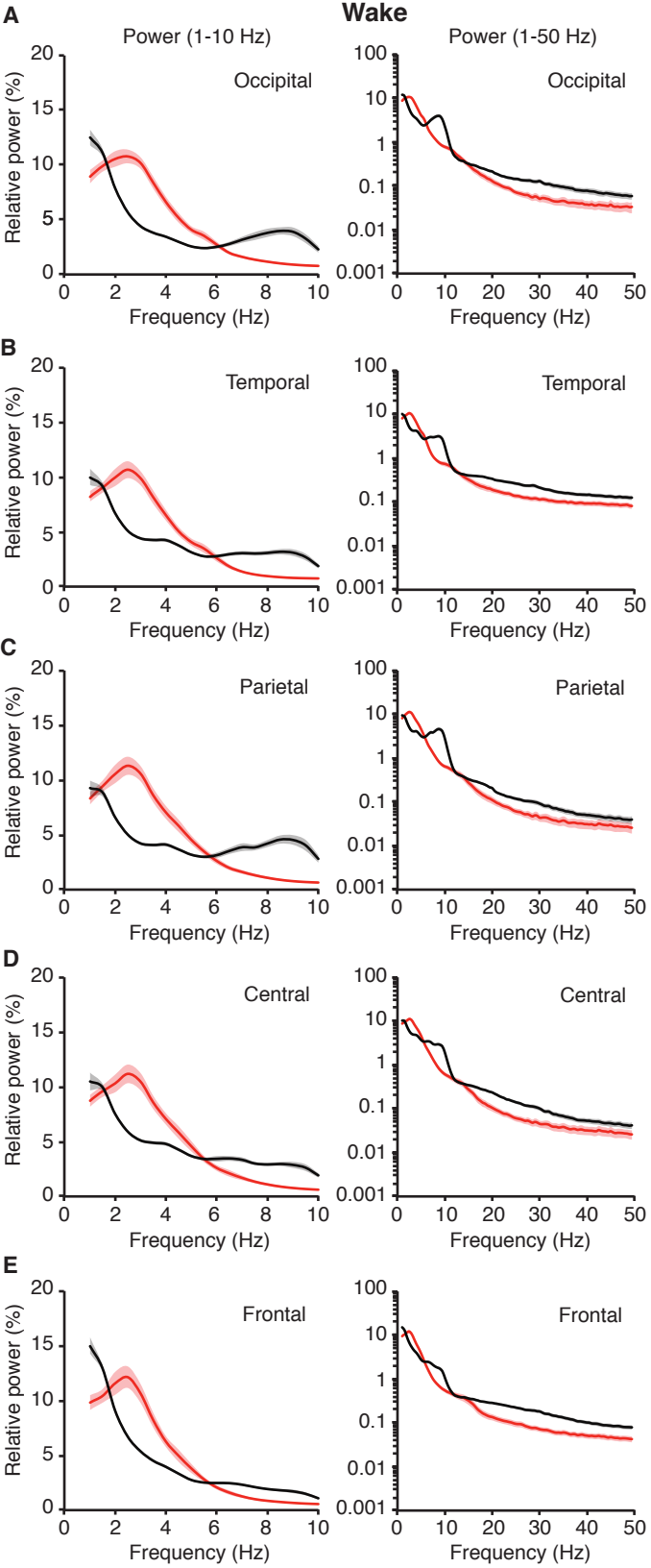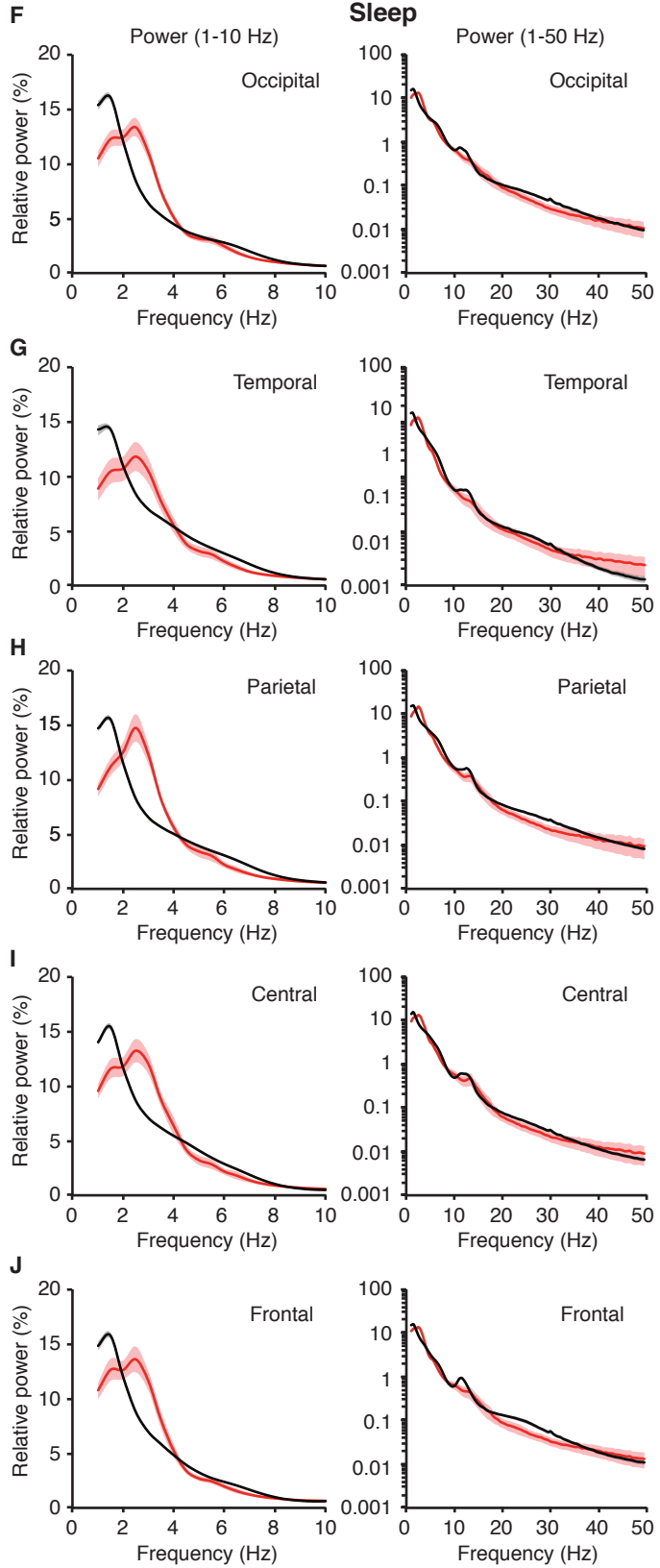

Supplement: Supplementary file 3 — Power spectra from all regions during epochs of wake and sleep. Black: neurotypical (NT), red: AS. During wakefulness (NT: n = 54, AS: n = 26), (A) occipital, (B) temporal, (C) parietal, (D) central, and (E) frontal spectra. During sleep (NT: n = 54, AS: n = 13), (F) occipital, (G) temporal, (H) parietal, (I) central, and (J) frontal spectra. (PDF 896 kb) [file 11689_2017_9195_MOESM3_ESM.pdf]
