# Supplementary material for: Delta rhythmicity is a reliable EEG biomarker in Angelman syndrome: a parallel mouse and human analysis
Source: J Neurodev Disord. 2017 May 8;9:17. doi: 10.1186/s11689-017-9195-8 (PMC5422949; doi:10.1186/s11689-017-9195-8)
Supplement: Supplementary file 5 — Examples of EEG variants in children with Angelman syndrome. (A–C) Three examples of enhanced delta oscillations generalized across the neocortex. (D) An example of delta oscillations restricted to posterior electrodes. (E) An example of delta oscillations restricted to frontal electrodes. (F) An example of delta oscillations restricted to frontal electrodes over the left hemisphere. (G, H) Examples of notched delta. (I) An example of theta oscillations. (PDF 9020 kb) [file 11689_2017_9195_MOESM5_ESM.pdf]

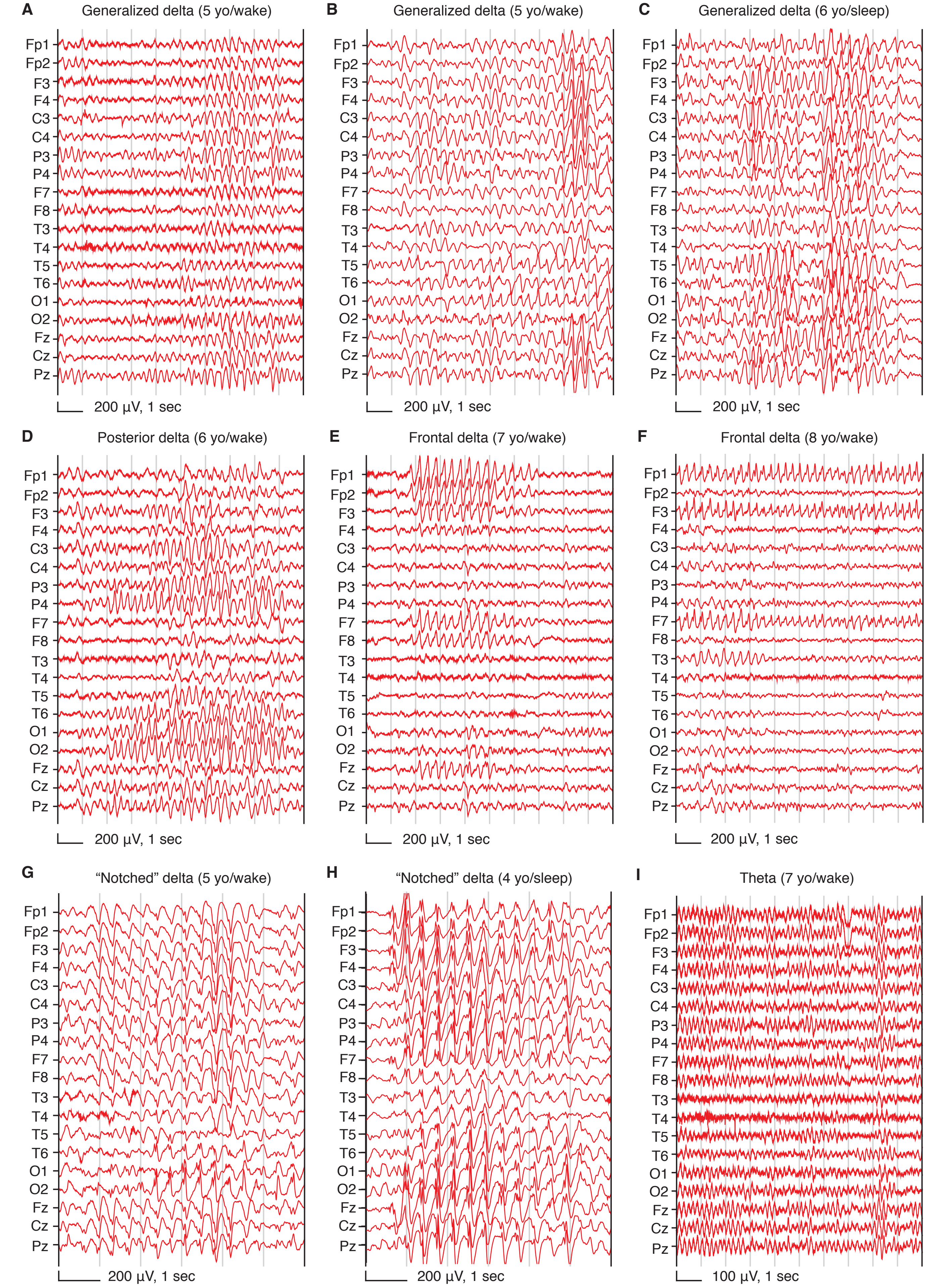

A
Generalized delta (5 yo/wake)
B
Generalized delta (5 yo/wake)
C
Generalized delta (6 yo/sleep)
D
Posterior delta (6 yo/wake)
E
Frontal delta (7 yo/wake)
F
Frontal delta (8 yo/wake)
G
"Notched" delta (5 yo/wake)
H
"Notched" delta (4 yo/sleep)
I
Theta (7 yo/wake)
Fp1 Fp2 F3 F4 C3 C4 P3 P4 F7 F8 T3 T4 T5 T6 O1 O2 Fz Cz Pz
200 µV, 1 sec
100 µV, 1 sec
