# Supplementary material for: Erratum to: Delta rhythmicity is a reliable EEG biomarker in Angelman syndrome: a parallel mouse and human analysis
Source: J Neurodev Disord. 2017 Jul 17;9:30. doi: 10.1186/s11689-017-9210-0 (PMC5514461; doi:10.1186/s11689-017-9210-0)
Supplement: Additional file 3: Figure S3. — Power spectra from all regions during epochs of wake and sleep. Black: neurotypical (NT), red: AS. During wakefulness (NT: n = 54, AS: n = 26), a occipital, b temporal, c parietal, d central, and e frontal spectra. During sleep (NT: n = 54, AS: n = 13), f occipital, g temporal, h parietal, i central, and j frontal spectra. (DOC 138 kb) [file 11689_2017_9210_MOESM1_ESM.doc]

**Fig S3 (Additional file 3)**


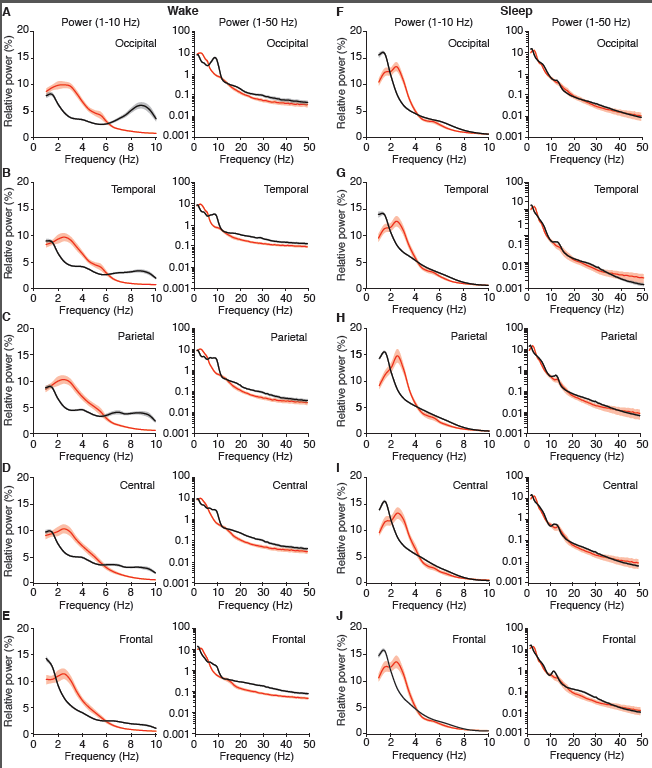


Power spectra from all regions during epochs of wake and sleep. *Black*: neurotypical (NT), *red*: AS. During wakefulness (NT: *n* = 54, AS: *n* = 26), **a** occipital, **b** temporal, **c** parietal, **d** central, and **e** frontal spectra. During sleep (NT: *n* = 54, AS: *n* = 13), **f** occipital, **g** temporal, **h** parietal, **i** central, and **j** frontal spectra.
